# Disrupting Mitochondrial–Nuclear Coevolution Affects OXPHOS Complex I Integrity and Impacts Human Health

**DOI:** 10.1093/gbe/evu208

**Published:** 2014-09-22

**Authors:** Moran Gershoni, Liron Levin, Ofer Ovadia, Yasmin Toiw, Naama Shani, Sara Dadon, Nir Barzilai, Aviv Bergman, Gil Atzmon, Julio Wainstein, Anat Tsur, Leo Nijtmans, Benjamin Glaser, Dan Mishmar

**Affiliations:** ^1^Department of Life Sciences, Ben Gurion University of the Negev, Beer Sheva, Israel; ^2^Institute of Aging, Division of Endocrinology, Departments of Medicine and Genetics, Albert Einstein College of Medicine, New York, NY, USA; ^3^Department of Systems and Computational Biology, Albert Einstein College of Medicine, New York, NY, USA; ^4^Diabetes Unit, E. Wolfson Medical Center, Holon, Israel; ^5^Endocrine Clinic, Clalit Health Services, Jerusalem, Israel; ^6^Nijmegen Center for Mitochondrial Disorders, Radboud University Medical Centre, Nijmegen, The Netherlands; ^7^Endocrinology and Metabolism Service, Hadassah-Hebrew University Medical Center, Jerusalem, Israel

**Keywords:** coevolution, complex I, mitonuclear interaction, mitochondria, mtDNA, *NDUFC2*

## Abstract

The mutation rate of the mitochondrial DNA (mtDNA), which is higher by an order of magnitude as compared with the nuclear genome, enforces tight mitonuclear coevolution to maintain mitochondrial activities. Interruption of such coevolution plays a role in interpopulation hybrid breakdown, speciation events, and disease susceptibility. Previously, we found an elevated amino acid replacement rate and positive selection in the nuclear DNA-encoded oxidative phosphorylation (OXPHOS) complex I subunit *NDUFC2*, a phenomenon important for the direct interaction of *NDUFC2* with the mtDNA-encoded complex I subunit *ND4*. This finding underlines the importance of mitonuclear coevolution to physical interactions between mtDNA and nuclear DNA-encoded factors. Nevertheless, it remains unclear whether this interaction is important for the stability and activity of complex I. Here, we show that siRNA silencing of *NDUFC2* reduced growth of human D-407 retinal pigment epithelial cells, significantly diminished mitochondrial membrane potential, and interfered with complex I integrity. Moreover, site-directed mutagenesis of a positively selected amino acid in *NDUFC2* significantly interfered with the interaction of *NDUFC2* with its mtDNA-encoded partner *ND4*. Finally, we show that a genotype combination involving this amino acid (*NDUFC2* residue 46) and the mtDNA haplogroup HV likely altered susceptibility to type 2 diabetes mellitus in Ashkenazi Jews. Therefore, mitonuclear coevolution is important for maintaining mitonuclear factor interactions, OXPHOS, and for human health.

## Introduction

The mitochondrion is the major source of cellular energy and its function is critical to the life of higher organisms. Mitochondria is especially important for the routine function of energetic tissues, such as the central and peripheral nervous systems, skeletal muscle, heart, liver, and pancreas (Zeviani and Di Donato 2004; Schapira 2006). Early in eukaryotic evolution, the once independently living mitochondrial ancestor lost most of its coding sequences to the host (Gray 2012). As a result, most of the ∼1,500 genes required for mitochondrial function (in humans) are encoded by the nucleus, whereas 37 genes are encoded by the maternally inherited circular mitochondrial DNA (mtDNA) (Calvo and Mootha 2010). The latter includes genes for 13 proteins of the adenosine three phosphate (ATP)-producing oxidative phosphorylation (OXPHOS) system, 2 rRNA genes (*12S, 16S*), and 22 tRNA genes. The protein-coding genes include those directing the synthesis of seven subunits (*ND1–6, ND4L*) of the NADH ubiquinone oxidoreductase (complex I), one subunit (*Cyt b*) of the ubiquinol cytochrome *c* reductase (complex III), three subunits (*CO1–3*) of the cytochrome *c* oxidase (complex IV), and two subunits (*ATP6,8*) of the F1-F0 ATP synthase (complex V). Given that mitochondria play a central role in energy production, it is not surprising that mitochondrial dysfunction affects cell metabolism (Ritov et al. 2010).

The fast rate of mtDNA change imposes tight coevolution of closely interacting mtDNA-encoded factors and nuclear DNA (nDNA)-encoded factors (Grossman et al. 2004; Bar-Yaacov et al. 2012; Dowling 2014). Accordingly, it was shown that interfering with mitonuclear coevolution leads to hybrid breakdown and reduces fitness due to OXPHOS dysfunction (Burton et al. 2006; Lee et al. 2008; Barreto and Burton 2013; Hoekstra et al. 2013; Meiklejohn et al. 2013). This led us to propose that mitonuclear incompatibility played a major role in the creation of reproductive barriers and even speciation events (Gershoni et al. 2009). In addition to the involvement of mitonuclear epistatic interactions in long-term evolutionary processes, it has been shown that certain mtDNA–nDNA genotype combinations affect the penetrance of genetic disorders (Johnson et al. 2001; Hudson et al. 2005; Potluri et al. 2009) and longevity (Zhu et al. 2014). Because most mitonuclear interactions occur between OXPHOS complex I components, there is major interest in assessing the involvement of such interactions in disease.

Several pieces of evidence underline the importance of complex I mitonuclear interactions in evolution and disease. First, cells harboring mitochondria from chimpanzee or gorilla and human nuclei display a specific ∼40% reduction in complex I activity (Barrientos et al. 1998). Second, Potluri et al. (2009) found that the phenotypic impact of a mutation in the nDNA-encoded subunit *NDUFA1* that causes progressive complex I-specific neurodegenerative disease was modulated by mtDNA genetic backgrounds (Potluri et al. 2009). Accordingly, *NDUFA1* was found to directly interact with two complex I mtDNA-encoded subunits (Gershoni et al. 2010). Third, Rai et al. (2007) found that a combination between the A10398G nonsynonymous change in mtDNA-encoded complex I subunit *ND3* with nDNA-encoded variants increased susceptibility to developing type 2 diabetes mellitus (T2DM). Finally, we found that genetic association of T2DM with the mtDNA genetic background haplogroup J1, which is mainly defined by nonsynonymous changes in complex I subunits, was modulated by nuclear genetic factors (Feder et al. 2009). Therefore, interfering with the mitonuclear interactions within complex I might play a role in disease, in general, and in T2DM, in particular. In addition to the evidence presented above, a genetic variant within the nDNA-encoded *NDUFB6* subunit of complex I was associated with altered methylation and gene expression patterns in insulin-resistant individuals (Ling et al. 2007). Finally, it has been shown that the antidiabetic agents, the thiazolidinediones, such as metformin, inhibit respiratory complex I (Brunmair et al. 2004). Hence, elucidating the structure and function of complex I and the interactions among its subunits are likely to shed light on the mechanisms underlying mitochondrial involvement in metabolic disorders, in general, and in T2DM, in particular.

Despite many years of efforts, the structure and network of subunit interactions within mammalian complex I remain only partially resolved (Vogel et al. 2007), although recent single particle analysis added much information (Vinothkumar et al. 2014). Although high-resolution crystal structures of complex I from bacteria (Efremov and Sazanov 2011) and the yeast *Yarrowia lipolitica* (Hunte et al. 2010) have shed light on such interactions, evolutionary distance still leaves the network of subunit interactions in mammalian complex I unresolved. By rigorous sequence analysis of nDNA-encoded subunits of complex I in primates, we identified three subunits (*NDUFA1, NDUFA4*, and *NDUFC2*) that exhibited accelerated amino acid rate of change, suggesting their candidacy as coevolving with the fast evolving mtDNA-encoded subunits (Mishmar et al. 2006). Recently, using a combined bioinformatics and molecular biology approach, we predicted and experimentally verified the interactions of two complex I nDNA-encoded subunits (i.e., *NDUFC2* and *NDUFA1*) with their coevolving mtDNA-encoded subunit partners (Mishmar et al. 2006; Gershoni et al. 2010). However, recent evidence has suggested that *NDUFA4*, the third subunit exhibiting an accelerated mutation rate, is a member of OXPHOS complex IV rather than of complex I, thus excluding this subunit from further analysis of mitonuclear interactions in the frame of complex I (Balsa et al. 2012).

What might be the physiological importance of mitonuclear coevolution in complex I? Remarkably, genetic variants in close linkage with *NDUFC2*, a subunit which physically interacts with the mtDNA-encoded complex I subunit *ND4*, were recently associated with decreased glucose-stimulated insulin secretion (Olsson et al. 2011). Furthermore, *NDUFC2* was identified as one of five complex I subunits, the expression levels of which were significantly reduced in insulin-resistant human skeletal muscle samples (Lefort et al. 2010). Taken together, these findings led us to hypothesize that mitonuclear interactions involving *NDUFC2* are important for mitochondrial function during evolution and in disease conditions.

Here, we tested our hypothesis by assessing the effect of *NDUFC2* silencing in human cells on mitochondrial function, cell life, and complex I integrity. Because *NDUFC2* underwent positive selection, we assessed the effect of directed mutagenesis of a positively selected amino acid within *NDUFC2* on the interaction with its mtDNA-encoded partner *ND4*. Finally, we assessed the association of T2DM with mitonuclear genotype combinations, including *NDUFC2* and mtDNA genetic backgrounds in Ashkenazi Jews.

## Materials and Methods

### Cell Line and Cultures

Human D-407 retinal pigment epithelium cells, a kind gift from R.C. Hunt (Davis et al. 1995), were grown using a standard CO_2_ cell culture incubator in DMEM medium supplemented with 3% fetal calf serum (FCS), 2 mM l-glutamine, 1,000 U/ml penicillin, and 1 mg/ml streptomycin.

### Design of siRNA Silencing of *NDUFC2* in Human Cells

The siRNA target sequence was designed using the Dharmacon siDESIGN website (http://www.dharmacon.com/DesignCenter/DesignCenterPage.aspx, last accessed September 25, 2014). Specifically, the siRNA oligonucleotide was designed using the 3′ untranslated region sequence of *NDUFC2*. The following commercial controls (Dharmacon) for transient knock-down were used: 1) siRNA agent against GAPDH (siGAPDH), as a positive control for the silencing protocol; 2) scrambled siRNA, as a negative control; and 3) Cy3 fluorescently labeled siRNA—siGLO, to assess transformation efficiency by fluorescence-activated cell sorter (FACS) (see details below).

### Transformation of siRNA into D-407 Cells

About 96 h prior to transformation, frozen cells were thawed and transferred into a 25-ml flask. Approximately 24 h prior to transformation, the cells were harvested with trypsin, neutralized by medium, manually counted, and transferred into a 6-well plate (∼1.5 × 10^5^ per well) in the presence of 2.5 ml full growth medium (see Cell Line and Cultures). After 24 h, 0.25 ml of the growth medium was drawn, and the transformation mixture was prepared as follows: 12 ml TransIT-TKO transformation reagent (Mirus) was added to 0.25 ml serum-free DMEM medium (Biological Industries, Bet Ha’emek, Israel) and mixed by pipetting. siRNA oligonucleotides were added to a final concentration of 25 nM and the mixture was incubated at room temperature for 5–20 min. The transformation medium was slowly added to the wells and incubated for 72 h in a CO_2_ incubator under standard growth conditions.

### Analysis of the siRNA-Treated Cells

siRNA-treated cells were examined 72 h posttransformation under an inverted phase microscope (Nikon) and photographed. The cells were then harvested and counted (per well). siRNA transformation efficiency was evaluated by FACS, comparing the ratio of cells that were transformed with Cy3-labled siGLO-labeled siRNA (Dharmacon) with untreated cells. Specifically, D-407 cells were harvested 14 and 72 h after transformation using trypsin/ethylenediaminetetraacetic acid. The samples were analyzed using a FACSCalibur flow cytometer (Becton Dickinson, San Jose, CA). The FLH2 filter was used to estimate the red emission of Cy3. Data were collected and analyzed using CellQuest Pro 4.0.2 analysis software.

### Estimating Silencing Efficiency

Total RNA was extracted from untreated or siRNA-treated cells following the Gentra Versagene total RNA purification kit protocol. cDNA was produced using a Bio-Rad iScript cDNA Synthesis kit, according to the manufacturer’s protocol. cDNA (500 nM, with concentration being determined using an Eppendorf Biophotometer) was used as template for a separate real-time polymerase chain reaction (PCR) amplification that was performed in a volume of 20 μl containing 1× ABsolute Blue SYBR Green ROX Mix (Thermo), with either of the following primer sets (50 nM of each primer): *NDUFC2* primers (forward: 5′-GG TTT GCA TCG CCA GCT TC-3′; reverse: 5′-CAG GAA AAT CCT CTG GAT G-3′) and ß-actin (forward:5′-CGC GAG AAG ATG ACC CAG AT-3′; reverse: 5′-TCA CCG GAG TCC ATC ACG AT-3′). The following PCR protocol was used in a Stratagene Mx3000P real-time PCR machine: 15 s at 94 °C followed by 40 cycles of denaturation for 10 s at 98 °C, annealing for 20 s at 60 °C and extension for 15 s at 72 °C. These cycles were followed by a final extension step of 7 min at 72 °C. The threshold cycle (Ct) values were derived from a standard curve generated with the aforementioned primers on commercial testis RNA (Ambion). For the real-time PCR experiment, RNA extracted from nontargeting siRNA transformed cells (negative control) was used as a calibrator tissue and ß-actin served as a normalizing gene.

### Blue Native Polyacrylamide Gel Electrophoresis Analysis

D-407 cells were treated by *NDUFC2* siRNA and a nontargeting control reagent, as described above. Forty-eight hours posttransformation, the cells were harvested using trypsin and washed twice with phosphate buffered saline (PBS). Following removal of residual PBS, the cells were immediately frozen in liquid nitrogen and kept on dry ice until further analysis by blue native polyacrylamide gel electrophoresis (BN-PAGE), performed as described previously (Calvaruso et al. 2008). Lanes were loaded with 80 μg of solubilized mitochondrial protein. After electrophoresis, the gels were further processed for in-gel complex I activity staining or western blotting. Protein immunodetection was performed using anti-*SDHA* (Mito Sciences), anti-*NDUFA9*, and anti-CORE 2 (Invitrogen) primary antibodies. Peroxidase-conjugated secondary antimouse or antirabbit IgG antibodies (Invitrogen) were then used. Antibody binding was detected using ECL plus (Amersham Biosciences).

### Membrane Potential Analysis

D-407 cells were grown in medium containing either 100% glucose (4.5 g/l) or in medium in which 80% or 90% of the glucose was replaced by galactose, added as an alternative carbon source. Forty-eight hours post-siRNA treatment, tetramethylrhodamine methyl ester (TMRM) was added to the wells to a final concentration of 500 nM, as previously described (Maldonado et al. 2010) and incubated for 30 min followed by PBS washing and cells harvesting. The cells were then centrifuged, resuspended twice with 200 µl PBS, plated on 96-well plates (Greiner bio-one, white, 655098), and analyzed for TMRM florescence using an enzyme-linked immunosorbent assay (ELISA) reader (excitation 488 nm; emission 573 nm).

### Site-Directed Mutagenesis at *NDUFC2* Amino Acid Position 46

Human mtDNA-encoded *ND4* (previously recoded to the cytoplasmic genetic code; Gershoni et al. 2010) and nDNA-encoded *NDUFC2* genes were cloned into the pBD and pAD plasmids of the yeast two-hybrid assay, as previously described. To alter *NDUFC2* amino acid position 46, we utilized our previously cloned pAD–*NDUFC2* plasmid (Gershoni et al. 2010) encoding the common leucine variant at amino acid position 46 as template. For site-directed mutagenesis, we used the Phusion site-directed mutagenesis protocol (Finnzyme) with the following primers: 1) *NDUFC2* L>V change: forward: 5′-GCCTGATTGATAACGTTATCCGGCGGAGGCCG-3′, reverse: 5′-CGGCCTCCGCCGGATAACGTTATCAATCAGGC-3′; 2) *NDUFC2* L>F change: forward: 5′-GGCCTGATTGATAACTTTATCCGGCGGAGGCCG-3′, reverse: 5′-CGGCCTCCGCCGGATAAAGTTATCAATCAGGCC-3′; and 3) *NDUFC2* V>A change: forward: 5′-CTGATTGATAACGCTATCCGGCGGAGG-3′, reverse: 5′-CCTCCGCCGGATAGCGTTATCAATCAG-3′. The reaction conditions used were according to manufacturer’s instructions. Briefly, the reaction program included 18 cycles of annealing at 55 °C for 30 s, 9 min extension at 72 °C, followed by Dpn1 treatment in the PCR buffer used and transformation into the DH5-α *Escherichia coli* host strain. The selected colonies were incubated at 37 °C for 12 h in 10 ml LB supplemented with 100 µg/ml ampicillin. Plasmid DNA was purified using the Wizard plus SV minipreps DNA purification system (Promega), according to the manufacturer’s protocol. The insert was sequenced at the Ben-Gurion University DNA sequencing facility.

### Yeast Two-Hybrid Assay

Yeast two-hybrid analysis was performed using the Yeast Two-hybrid Phagemid vector kit (Stratagene). The pAD–*NDUFC2*-L (wild type, WT) or other pAD–*NDUFC2* mutant plasmids (*NDUFC2*-V, *NDUFC2*-A, and *NDUFC2*-F) were used as bait, while a plasmid encoding pBD–*ND4* was used as prey. The PJ yeast host strain was cotransformed with plasmids pAD–*NDUFC2* WT (*NDUFC2*-L) or the mutants and pBD–*ND4* plasmids using the LiAC method. Single transformants were grown in liquid SC-Leu-Trp to OD_600_ 10, washed twice with double distilled water (DDW), and diluted to an initial OD_600_ of 0.3. For quantitative two-hybrid analysis, the generation time of the indicated mutants and their respective partner, *ND4*, were calculated from their growth curves in liquid SC-Leu-Trp-His media. Cells were grown overnight in SC-Leu-Trp, washed twice with DDW, diluted 1:10 into 5 ml of prewarmed SC-Leu-Trp-His, and OD_600_ measurements were taken. Generation times (*t*) were calculated from the growth curves according to the equation OD*_t_* = OD_0_ × 2*^t^*^/^*^T^*. The generation time calculated for each culture is an average of at least four independent experiments. Student’s *t*-test was performed on the results obtained from at least four independent experiments to assess significance.

### Western Blot Analysis

In order to assess protein expression of the fused pBD construct, we extracted proteins from yeast, loaded them on a 10% polyacrylamide gel, and analyzed them by western blot exactly as previously described (Gershoni et al. 2010) utilizing rabbit polyclonal antibodies raised against the Gal4-binding domain (Santa Cruz Biotechnology, a generous gift from Prof. Michal Shapira, BGU). This procedure was applied to each independent repeat of the yeast two-hybrid assay (four repeated experiments). Although we attempted to perform the reciprocal experiment using two commercial antibodies raised against NDUFC2 (ABCAM and ADAR technology LTD, respectively), in our hands control extract from cells did not result in a band with the expected size. In addition, an antibody raised against pAD (Santa Cruz Biotechnology, a generous gift from Prof. Michal Shapira, BGU) did not result in a clear band. Therefore, we excluded these antibodies from the current work.

### Sequence Alignment and Positive Selection

**Fig. 1.—:**
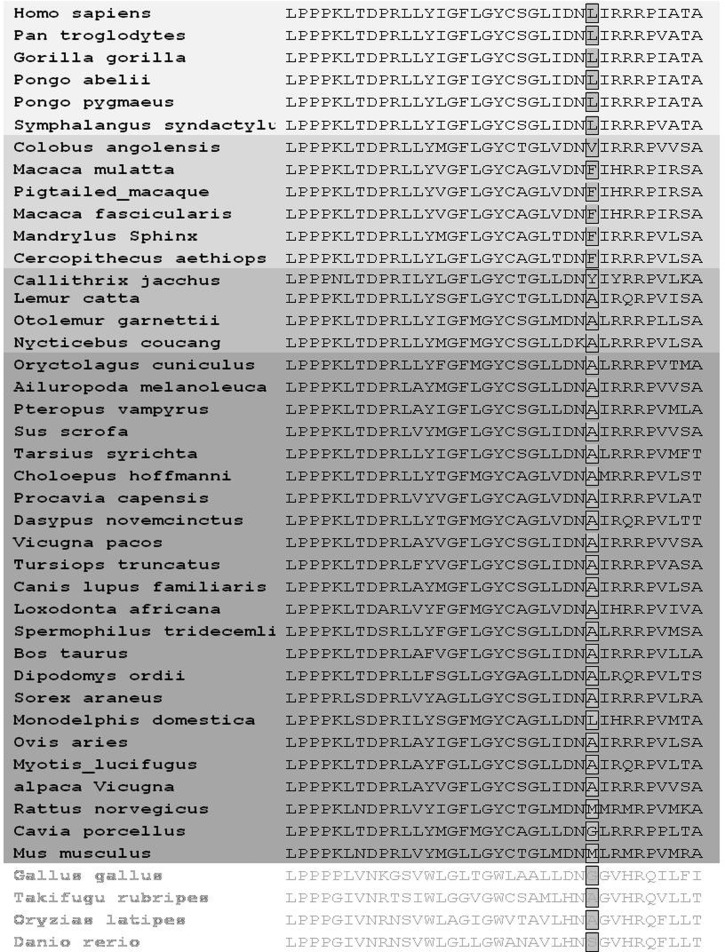
Phylogenetic analysis of *NDUFC2* at the region encoding amino acid position 46 and flanking sequences. NDUFC2 position 46 is highlighted. Ape, old world monkeys, prosimians, and nonprimate mammalian sequences are presented on a gray-scale gradient running from light to dark gray, respectively.

*NDUFC2* cDNA sequences available from GenBank, as well as the deduced proteins encoded by open reading frames from 39 mammals and 4 nonmammalian vertebrates, were aligned using ClustalW and T-Coffee. Because both methods resulted in consistent alignments, we continued using the ClustalW results for the sake of simplicity. Those species for which *NDUFC2* sequences were available are listed in figure 1. The cDNA sequence alignment was further utilized for the analysis of positive selection using SELECTON (selecton.tau.ac.il) (Stern et al. 2007), as described (Gershoni et al. 2010). Briefly, the tested hypothesis is whether positive selection is operating on *NDUFC2*, as contrasted with a null hypothesis which assumes that there is no positive selection. The SELECTON server allows for detecting selective forces even at single amino acid sites. The ratio of nonsynonymous (i.e., amino acid altering) to synonymous (i.e., silent) substitutions, known as the Ka/Ks ratio, is used to estimate both positive and purifying selection at each amino acid site.

### The Tested Cohort

DNA samples from individuals of Ashkenazi Jewish origin were collected by the Israeli Diabetes Research Group. The patient study group was composed of 1,118 individuals comprising the following: 1) 719 unrelated T2DM patients (360 men and 359 women; average age 65.8 years) whose DNA samples were previously analyzed (Feder et al. 2008, 2009) and 2) 399 new individuals described in this study. The 719 patients were already analyzed for their mtDNA genetic backgrounds using restriction fragment length polymorphism (RFLP) sites throughout the mtDNA sequence, which were reported along with additional clinical details. Because of DNA sample limitations, we utilized only a subset of the previously studied patient cohort. Blood samples from all patients were collected at seven different medical centers throughout Israel and DNA was extracted using standard techniques (Puregene; Gentra Systems, Minneapolis, MN). Similar to the previous (719) patient group, the new 399 T2DM patients were also family unrelated. A control cohort comprised two groups: 1) 170 individuals (89 men and 81 women; average age 69.7 years) who were previously analyzed for their mtDNA genetic backgrounds (Feder et al. 2007). All individuals gave written informed consent prior to sample collection. The study was approved by the “The Committee on Research Involving Human Subjects of the Hebrew University-Hadassah Medical School” (application approval number 36-03.09.04) and by the local Institutional Review Boards of each participating institution. 2) An additional group of 189 healthy Ashkenazi Jews (77 men and 112 women; average age 68.6 years) were collected at the Albert Einstein College of medicine. Informed written consent was obtained in accordance with the policy of the Committee on Clinical Investigation of the Albert Einstein College of Medicine. In summary, 1,118 unrelated T2DM patients and 359 healthy controls were used in this study (supplementary table S1, Supplementary Material online).

### mtDNA Haplogroup Assignment

Part of the patient samples (*N* = 719) and from controls (*N* = 170) were previously analyzed for mtDNA haplogroup affiliation using PCR-RFLP of selected mtDNA markers (Feder et al. 2007, 2008, 2009). Genotype information for the 420 patients added in this study was obtained using the MetaboChip single nucleotide polymorphism (SNP) chip which harbors 194,263 nDNA SNPs and 126 mtDNA SNPs (http://www.illumina.com/, last accessed September 25, 2014). These mtDNA SNPs were utilized for detailed mtDNA haplogroup assignment using HaploGrep (Kloss-Brandstätter et al. 2011). Three hundred and ninety-nine patients (204 men and 195 women; average age 62.8 years) of the initial 420 patients had sufficient SNP information to allow assignment to one of the four haplogroup clusters (HV, UK, IW, and JT). The control group (*N* = 359) was analyzed for their mtDNA haplogroup affiliation as follows: 170 healthy controls were previously analyzed for their mtDNA haplogroup affiliation (Feder et al. 2007). The additional healthy controls (*N* = 189) were genotyped using the Affymetrix 6.0 SNP chip (http://www.affymetrix.com, last accessed September 25, 2014). The chip used harbored 906,481 nDNA SNPs and 119 mtDNA SNPs, of which 28 were informative in our analyzed population for mtDNA haplogroup assignment in HaploGrep (supplementary table S2, Supplementary Material online) (Kloss-Brandstätter et al. 2011). Haplogroup distribution in all patients and controls is reported in table 1.

**Table 1:** Mitochondrial DNA Haplogroup Distribution in Patient and Control Groups

mtDNA Haplogroup Cluster	Patient	Control	Proportion of Patients
HV	394 (35.2%)	145 (40.4%)	0.731
UK	431 (38.6%)	128 (35.7%)	0.771
JT	163 (14.6%)	43 (12%)	0.791
IW	130 (11.6%)	43 (12%)	0.751
Total	1,118	359	0.757

Note.—The actual number of individuals is listed, while the percentages of each haplogroup cluster in each of the studied groups are presented in parentheses. The proportion of patients in each haplogroup cluster is also listed.

### Amplifying and Sequencing the Coding Region of *NDUFC2*

PCR exon amplification of *NDUFC2* was performed using DNA samples as templates and forward and reverse primers encompassing the sequence of each exon (supplementary table S3, Supplementary Material online). To identify common variants (present at a frequency >1%) within our studied populations, *NDUFC2* exons were PCR amplified. For PCR amplification during the genotyping process, we used 50 ng of DNA from each sample (collected from 25 randomly chosen, healthy Ashkenazi Jews) as template in a standard 20 μl PCR assay containing 250 nM dNTPs (Bio-Lab, Jerusalem, Israel), 10 nM of each primers (IDT, Jerusalem, Israel), 1 U of Taq-polymerase (Bio-Lab), varying concentration of MgCl_2_ (supplementary table S4, Supplementary Material online), and 10× Taq Polymerase (TAQ) reaction buffer (Bio-Lab). The PCR program included a denaturation stage of 4 min (94°C) followed by 30 cycles of denaturation at 94 °C, annealing for 30 or 60 s at different temperatures according to the calculated temperature of melting (TM) of the primers employed (supplementary table S4, Supplementary Material online), extension at 72 °C, a final extension of 5 min at 72 °C, and a concluding step in which the reactions were cooled to 10 °C. The products were visualized on a 1.5% agarose gel stained with ethidium bromide and stored at −20 °C until use. The specific conditions for each reaction are summarized in supplementary table S4, Supplementary Material online. After amplification, the samples were cleaned with a DNA purification kit (Promega) and sequenced by the Applied Biosystems (ABI) PRISM 3100 Genetic Analyzer at the Ben Gurion University DNA sequencing core facility.

### Polymerase Chain Reaction Restriction Fragment Length Polymorphism

A common amino acid replacement variant was identified in exon 1 of *NDUFC2* (rs8875) in our tested population (Ashkenazi Jews). To screen for the allele distribution of this variant in our studied populations, a 326-bp *NDUFC2* exon 1 segment was PCR amplified using forward (5′-CTT ACG GTT TCT GCC GGA TG-3′) and reverse (5′-TGA CAC TCT GGC TCT GCT TG-3′) primers. For restriction digestion of the amplified sequence, the enzyme HpyCH4IV, which recognizes the ACGT/TGCA motif, was used. Each digestion reaction (total volume of 20 μl) contained 2 μl buffer, 0.5 μl enzyme, 12.5 μl DDW, and 5 μl PCR product. Digestion was performed at 65 °C for 1 h. As the enzyme does not recognize the common C variant but rather the rare G variant, the restriction digest was expected to yield 215 and 111 bp bands. This assay was applied to the entire patient and control cohorts.

### Statistical Analysis

We first used an *R* × *C* (rows × columns) test of independence to compare the distribution of the four mtDNA genetic backgrounds (i.e., haplogroup clusters HV, UK, JT, and IW) and three nDNA *NDUFC2* genotypes (i.e., CC, CG, and GG) among our T2DM patients and controls. Next, to detect candidate mtDNA–nDNA genotype combinations differing in their representation between the patients and controls, we used a permutation test. Specifically, we divided the tested samples into 12 different mtDNA–nDNA genotypes (4 mtDNA lineages × 3 nDNA *NDUFC2* genotypes = 12 mtDNA–nDNA genotypes; table 2). The diabetic status (a binary indicator variable, with 0 representing healthy individuals and 1 representing T2DM patients) was randomly shuffled among all samples. Then, the proportion of T2DM samples in each of the mtDNA–nDNA genotypes was calculated, and the absolute difference (two-tailed test) between each of these values and the general tendency to develop the disease in the entire population was recorded. This procedure was repeated 10,000 times. *P* values were estimated as the proportion of cases in which the absolute difference obtained during the simulation was equal to or greater than that of the original data set. The test was performed using MATLAB script. Finally, we used logistic regressions to assess whether the susceptibility of developing T2DM (represented by a binary indicator variable taking on values 0 and 1) differed among mtDNA–nDNA genotypes, while adjusting for individual characteristics, including gender and age. By converting the categorical variable “mtDNA–nDNA genotype” into binary numbers, we compared the candidate mtDNA–nDNA genotype detected using the permutation test (i.e., HV–CC) with each of the other 11 mtDNA–nDNA genotypes using a single test. Because this variable is composed of 12 classes, its inclusion in a logistic regression model requires generating 11 indicator variables. The coefficients of these indicator variables indicate whether the propensity to develop T2DM in each of the respective mtDNA–nDNA genotypes differs from that of the reference genotype (i.e., HV–CC). To obtain an estimate of the relative risk of individuals belonging to a particular mtDNA–nDNA genotype to develop T2DM, odds ratios (ORs) were calculated. To correct for multiple testing in the logistic regression and the permutation test, we estimated *q* values. Specifically, q values (measuring the minimum false discovery rate, which is incurred when calling a test significant) were estimated using the smoother method proposed by Storey and Tibshirani (2003) and the bootstrap method proposed by Storey (2002) and Storey et al. (2004), both implanted in the QVALUE software package. Student’s *t*-test was used to estimate differences between mitochondrial membrane potential measurements or cell counts in *NDUFC2*-silenced versus untreated cells, taking into account repeated measurements collected from independently repeated experiments (see Results).

**Table 2:** The Results of Permutation Tests Searching for Candidate mtDNA–nDNA Genotypes that Differ in Their Representation between Patients and Controls

mtDNA haplogroup–*NDUFC2* genotype combination	Diabetic	Total Number	Diabetic Proportion	*P* value	*q* Value (π_0_ = 0.227) Smoother	*q* Value (π_0_ = 0.208) Bootstrap
**HV_CC**	183	265	0.691	**0.006**	**0.016**	**0.015**
HV_CG	184	236	0.780	0.413	0.128	0.117
HV_GG	27	38	0.711	0.557	0.138	0.127
IW_CC	67	82	0.817	0.230	0.105	0.096
IW_CG	54	81	0.667	0.063	0.057	0.052
IW_GG	9	10	0.900	0.469	0.128	0.117
JT_CC	94	118	0.797	0.318	0.124	0.114
JT_CG	52	68	0.765	0.887	0.202	0.185
JT_GG	17	20	0.850	0.439	0.128	0.117
UK_CC	206	284	0.725	0.200	0.105	0.096
**UK_CG**	174	214	0.813	**0.036**	**0.049**	**0.045**
UK_GG	51	61	0.836	0.172	0.105	0.096
**Total**	1,118	1,477	0.757			

Note.—First column, left: The first two letters correspond to the mtDNA haplogroup cluster and the last two letters represent the *NDUFC2* genotype (in nucleotides). Numbers in the second and third columns are the actual numbers of samples. q values (measuring the minimum false discovery rate, which is incurred when calling a test significant) were estimated using the smoother method proposed in Storey and Tibshirani (2003) and the bootstrap method proposed in Storey (2002), both implanted in the QVALUE software package (Storey 2002). π_0_ measures the overall proportion of true null hypotheses.

### Web Resource

1. SELECTON server: http://selecton.tau.ac.il/
2. Predictprotein server: http://www.predictprotein.org/
3. Dharmacon siDESIGN website: http://www.dharmacon.com/DesignCenter/DesignCenterPage.aspx
4. HAPMAP server: www.hapmap.org
5. dbSNP server: www.ncbi.nlm.nih.gov/SNP (All URLs were accessed on September 25, 2014.)

## Results

### Assessing the Functional Role of *NDUFC2* in Cells

We previously showed that *NDUFC2*, and the mtDNA-encoded complex I subunit *ND4*, coevolved during primate evolution and interact physically. This interaction gained recent support from single particle analysis of mammalian complex I (Vinothkumar et al. 2014). Hence, we assessed the importance of *NDUFC2* for mitochondrial function, in general, and complex I activity, in particular. To assess the role played by *NDUFC2* in cell life and mitochondrial function, we employed siRNA to silence the expression of *NDUFC2*. Oligunucleotide transformation into human D-407 cells resulted in ∼90% reduction in *NDUFC2* transcript levels in siRNA-treated cells, as compared with cells treated by a nontargeting silencing agent (supplementary fig. S1, Supplementary Material online). Cell counting (performed in four independent experiments) 72 h posttreatment revealed significant reduction in the amount of *NDUFC2*-silenced cells, as compared with cells challenged with a nontargeting silencing agent in the D-407 cell line (*t*-test, *P* = 0.003) (supplementary fig. S2, Supplementary Material online). Similar results were obtained using HEK293 cells (data not shown). Hence, *NDUFC2* silencing reduced the growth rate of human cells.

### *NDUFC2* Is Important for OXPHOS Function

**Fig. 2.—:**
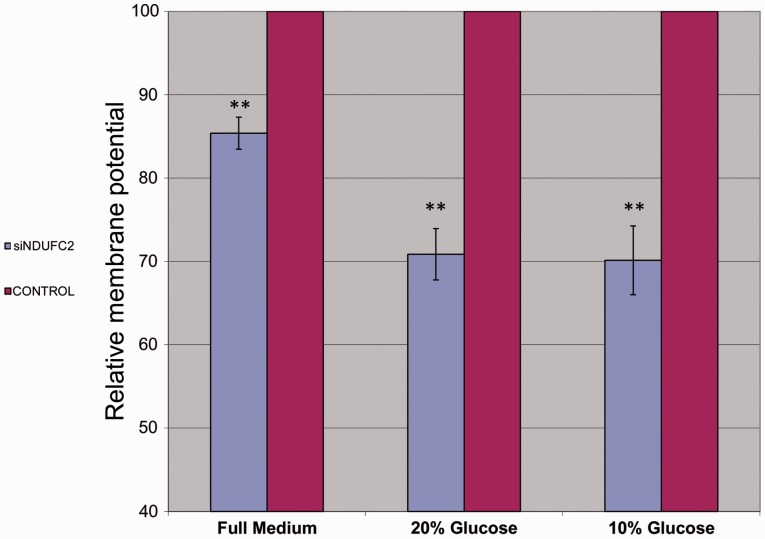
Relative quantification of mitochondrial membrane potential in the D-407 cell line 48 h after *NDUFC2* siRNA treatment. D-407 cells either treated by *NDUFC2*-silencing siRNA agent (blue columns) or by nontargeting siRNA agent (red columns) were grown either in full medium (4.5 g/l glucose) or in medium in which 80% or 90% of the glucose was replaced by galactose (i.e., 20% or 10% glucose-containing medium, respectively). Cells were incubated with 500-nM TMRM, an indicator of mitochondrial membrane potential, and fluorescence levels were measured by ELISA (see Materials and methods). *X* axis: In each of the experiments, column pairs are presented—a red column for the membrane potential measurements in nontargeting siRNA-treated cells and a blue column for the measurements in *NDUFC2* siRNA-treated cells. Three experiments are presented, differing in the cell growth media (all DMEM based): Full medium, 4.5 g/l glucose; 20% glucose, 0.9 g/l glucose, 3.6 g/l galactose; 10% glucose, 0.45 g/l glucose, 4.05 g/l galactose. Y axis: Membrane potential represented in arbitrary units (1–100). In each of the experiments, measurements obtained in cells treated with the nontargeting agent are considered as the maximum value (100 arbitrary units). **P < 0.001, two-tailed *t*-test.

To determine the importance of *NDUFC2* for OXPHOS activity, in general, and for OXPHOS complex I, in particular, we first measured mitochondrial membrane potential in D-407 cells by assessing the absorption of the tetramethylrhodamine methylester (TMRM), as previously described (Ehrenberg et al. 1988; Lemasters and Ramshesh 2007; Maldonado et al. 2010), 48 h after *NDUFC2* siRNA silencing. This analysis revealed a significant 15% reduction in the fluorescent signal within the siRNA-treated cells, relative to the nontargeting knock-down controls (three independent experiments, two-tailed *t*-test, *P* < 0.001). Because the use of galactose as a major carbon source in cell culture media is an established condition to drive cells into dependence on OXPHOS as a major source for ATP production (Reitzer et al. 1979; Bayona-Bafaluy et al. 2011), we grew *NDUFC2*-silenced cells and control cells either in full medium (i.e., with glucose as a carbon source) or in medium in which 80% or 90% of the glucose was replaced by galactose (i.e., 20% or 10% glucose-containing medium, respectively; see Materials and methods). Mitochondrial membrane potential (as assessed by TMRM measurements) was reduced by 30% in the *NDUFC2*-silenced cells, as compared with cells treated by nontargeting siRNA or control cells grown in galactose-based media (two-tailed *t*-test, *P* < 0.001) (fig. 2). These results indicate that *NDUFC2* is important for mitochondrial function.

### *NDUFC2* Silencing Affects Complex I Stability

**Fig. 3.—:**
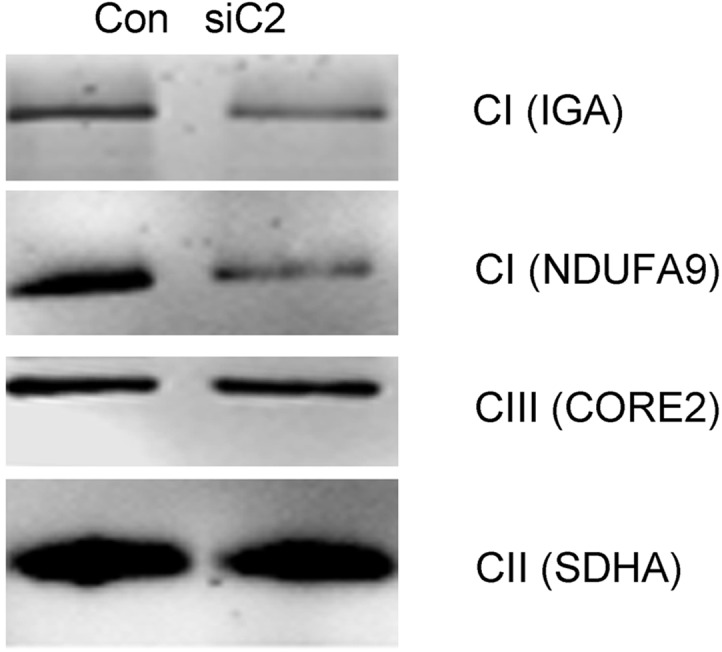
*NDUFC2* is required for complex I integrity. A representative experiment showing the analysis of total protein extracts by BN-PAGE. Con—protein extract from cells treated with a nontargeting siRNA control oligonucleotide; siC2—protein extract from cells treated with an *NDUFC2* siRNA oligonucleotide. Complex I in-gel activity staining assay and immune detection of complex I (*NDUFA9*), complex III (core II), and control CII 70 kDa were performed in protein extracts from control cells treated with nontargeting siRNA (left lane) or *NDUFC2*-targeting siRNA (right lane). A notable decrease in the amount of fully assembled complex I was identified, as was a decrease in complex I activity. CII—OXPHOS complex II.

Because we recently underlined the importance of *NDUFC2* for mitonuclear protein–protein interactions (Gershoni et al. 2010), we asked whether the function of *NDUFC2* is important for complex I assembly and stability. To this end, we used BN-PAGE to examine proteins extracted from D-407 cells that were treated either by *NDUFC2* siRNA or by nontargeting control siRNA. A notable decrease in the total amount of assembled complex I was observed in the *NDUFC2* siRNA treated cells. This is consistent with decreased NADH oxidation, which is mediated primarily by complex I, as measured by an in-gel activity stain assay using the artificial electron acceptor nitrotetrazolium blue in the siRNA-treated cells. The mobility of bands corresponding to complex I observed in all assays was identical in the control and siRNA-treated cells, indicating that the residual amount of complex I in the siRNA-treated cells likely corresponds to the fully assembled protein complex. We did not observe any subassembly intermediates representing degraded or partially assembled complex I in the siRNA-treated cells. These data suggest that *NDUFC2* indeed plays a role in the integrity (i.e., assembly and/or stability) of complex I (fig. 3 and supplementary fig. S3, Supplementary Material online).

### *NDUFC2* Amino Acid Position 46 Is Important for Mitonuclear Protein–Protein Interactions

**Fig. 4.—:**
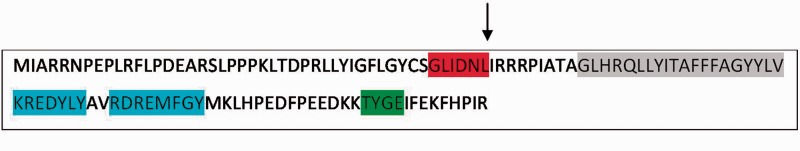
Graphic summary of NDUFC2 domain predictions. Red background, N-myristylation site and protein–protein interaction motif (http://www.predictprotein.org/); gray background, transmembrane domain; turquoise background, tyrosine kinase phosphorylation; green background, casein kinase II phosphorylation site. Arrow points to amino acid position 46.

As the first step toward identifying amino acid positions that are important for protein–protein interaction of *NDUFC2* with its mtDNA-encoded partner and their possible involvement in human health, we performed in silico sequence analysis. This analysis predicted that amino acid positions 41–46 likely constitute a protein–protein interaction domain (fig. 4). Within this putative domain, one amino acid (D44) was strongly negatively selected, whereas two others (I43 and L46) were positively selected during primate evolution (Gershoni et al. 2010), thus suggesting their functional potential. Only one of these amino acids, L46, also harbored a common human genetic variant (L46V, dbSNP accession number rs8875) which attracted our attention to its possible association with human genetic phenotypes (see below). Therefore, we focused our further analyses on the functional role of NDUFC2 amino acid position 46.

**Fig. 5.—:**
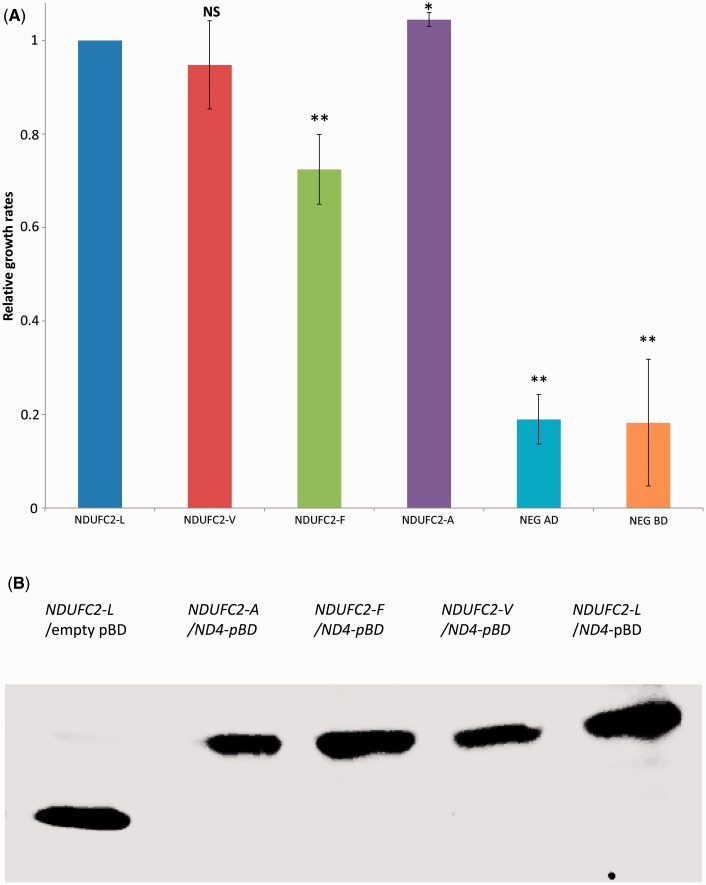
NDUFC2 amino acid position 46 is important for mitonuclear protein–protein interactions in a yeast two-hybrid assay. (*a*) Yeast growth rates, reflecting the levels of interaction of NDUFC2 with ND4. Yeast cells were transformed with the *ND4*–pBD construct in combination with either of the following constructs: *NDUFC2*-L (leucine), *NDUFC2*-V (valine), *NDUFC2*-F (phenylalanine), or *NDUFC2*-A (alanine). NEG-AD, negative control (plasmid ND4–pBD cotransformed with an empty pAD, NEG-AD). NEG-BD, negative control (plasmid *NDUFC2*–pAD cotransformed with an empty or pBD vector, NEG-BD). For assessing fold change in NDUFC2–ND4 interaction affinities, measurements of the generation rate of the yeast two-hybrid assay strains grown in selective liquid media lacking histidine were compared with cells expressing *NDUFC2*-L (see Materials and Methods). Significance is estimated relative to cells expressing wt *NDUFC2*-L. Fold increases or decreases in NDUFC2–ND4 interaction affinities were measured as the generation time of the different yeast two-hybrid strains grown in liquid selective media lacking histidine (Fridman et al. 2010). Significance—all measurements were compared with *NDUFC2*-L. *P = 0.02; **P < 0.001; NS, nonsignificant. (*b*) Western blot analysis of pBD expression. Gel loaded with protein extract from yeast transfected with (from left to right): 1) NDUFC2-L/empty pBD; 2) NDUFC2-A/ND4–pBD; 3) NDUFC2-F/ND4–pBD; 4) NDUFC2-V/ND4–pBD; 5) NDUFC2-L/ND4–pBD.

We anticipated that changes at a single amino acid position might only quantitatively alter the protein-binding capacity of NDUFC2. Previously, the yeast two-hybrid assay was adapted to measure yeast growth rate in selective liquid media, thus assessing affinity changes in protein–protein interactions in a semiquantitative manner (Fridman et al. 2010). To employ this same approach for our purposes, we cloned the mtDNA-encoded *ND4* gene, recoded to comply with the cytoplasmic genetic code, into a pBD plasmid, as previously described (Gershoni et al. 2010). To assess the importance of NDUFC2 position 46 for the interaction with ND4, we cotransformed the pBD–*ND4* construct into yeast along with each of four different *NDUFC2*–pAD constructs (A–D), encoding a different amino acid at position 46. The replacements within position 46 were planned to represent amino acid changes during mammalian evolution and human population genetic variants while retaining hydrophobicity. These included the following: 1) *NDUFC2*-L, encoding leucine at position 46, representing the first human allele; 2) *NDUFC2*-V, encoding valine at position 46, corresponding to the second human allele; 3) *NDUFC2*-A, encoding alanine at position 46, a small hydrophobic amino acid, representing the most abundant amino acid in nonprimate mammals (fig. 1); and 4) *NDUFC2*-F, encoding phenylalanine at position 46, a large hydrophobic amino acid, representing the most abundant amino acid in old world monkeys (fig. 1). Yeast growth rates, calculated from the growth curves of each experiment, demonstrated a significant ∼40% decrease in *NDUFC2*-F/*ND4* affinity, as compared with that of *NDUFC2*-L/*ND4* affinity (fig. 5*a*, *t*-test, *P* < 0.001). In contrast, a slight but significance increase in the *NDUFC2*-A/*ND4* growth rate was observed, as compared with that of *NDUFC2*-L/*ND4* growth rate (fig. 5*a*, *t*-test, *P* = 0.02). In order to assess whether the observed differences in protein–protein interaction is true or due to a lack of protein expression, we performed western blot analysis (fig. 5*b*). Close inspection revealed a possible difference in band intensities. Because each of the yeast growth rate experiments (fig. 5*a*) were repeated four times and each was accompanied by a western blot analysis (see Materials and Methods), we inspected these other blots. We found that the apparent band intensities’ differences did not correlate with the observed consistent differences in growth rates of the tested clones (fig. 5*a*). Because equal amounts of proteins were loaded onto the lanes in all western blots, we interpret the band intensities’ differences as reflecting technical variation among the blots, rather than true differences in protein expression levels. Hence, this analysis confirmed that the pBD construct was indeed expressed in the presence of all the tested proteins and mutants while growing the yeast in -L-W restrictive media, and, therefore, the observed differences in interaction levels are likely real. Taken together, these results support a functional role for NDUFC2 position 46 in mitonuclear protein–protein interactions. However, in our hands, no significant differences were observed while comparing the growth curves of yeast harboring *ND4*/*NDUFC2*-V or *ND4*/*NDUFC2*-L (fig. 5*a*). We, therefore, hypothesized that if the human genetic variants L46V affect mitonuclear interactions, such an effect should be subtle, below the sensitivity of our test, as well as many other available tests. To increase the sensitivity and overcome this obstacle, we explored an approach with higher sensitivity. To this end, we designed a disease-association study.

### NDUFC2 Amino Acid Position 46 Is the Only Position Harboring a Common Nonsynonymous Variant in Caucasians, in General, and Ashkenazi Jews, in Particular

Because amino acid position 46 proved important for NDUFC2 protein interaction with its mtDNA-encoded partner ND4, we hypothesized that natural genetic variants in this site should have phenotypic consequences. A screen of publicly available databases for nonsynonymous polymorphisms in *NDUFC2* (HAPMAP, www.hapmap.org [last accessed September 25, 2014]; dbSNP, www.ncbi.nlm.nih.gov/SNP [last accessed September 25, 2014]) revealed only a single validated nonsynonymous common variant—the one encoding amino acid position 46 (rs8875). This SNP yielded an L46V substitution, in which the leucine allele encompassed ∼70% and the valine allele comprised ∼30% of Caucasian genetic diversity. Because a linked SNP to *NDUFC2* altered the susceptibility to glucose-stimulated insulin secretion (Olsson et al. 2011), a diabetes-related phenotype, we asked whether the *NDUFC2* L46V SNP could alter susceptibility to diabetes. Because the T2DM patient cohort available to us was of Ashkenazi Jewish origin, and because bottlenecks could, in principle, alter the prevalence of rare variants in this population, we initially screened for the most common variants by sequencing all the coding *NDUFC2* exons in 25 healthy subjects. This screen confirmed the existence of the above-mentioned *NDUFC2* nonsynonymous variant, rs8875, and did not reveal any other amino acid replacements. As position 46 directly affected the interaction of NDUFC2 protein with its mtDNA-encoded partner ND4, we aimed toward assessing the association of genotype combinations involving this SNP and mtDNA SNPs with T2DM.

### A Mitonuclear Genotype Combination Involving NDUFC2 Position 46 Alters Susceptibility to Developing T2DM

We investigated the association of combinations of the *NDUFC2* amino acid replacement SNP rs8875 and mtDNA genetic backgrounds (haplogroups) with T2DM in patient and control cohorts of Ashkenazi Jewish origin. First, using PCR-RFLP, we assessed the allelic composition of the L46V *NDUFC2* variant in our cohort of 1,118 patients and 359 healthy controls. This screen revealed a similar allelic composition as seen in the general non-Jewish Caucasian population, that is, G (leucine allele):C (valine allele) was 0.74:0.26 in the controls and 0.7:0.3 in patients. In addition, we could not detect any significant differences in the distribution of the respective genotypes (i.e., CC, CG, and GG) among the patients and controls (*R* × *C* test of independence; *G* = 4.868, degrees of freedom [DF] = 2, *P* = 0.088).

Second, we assessed the association of mtDNA haplogroups with T2DM. To avoid sample size issues while comparing frequencies of mtDNA haplogroups, we grouped the mtDNA haplogroups according to phylogenetic considerations. This resulted in four merged haplogroup clusters: HV (haplogroups H and V), UK (haplogroups U and K), JT (haplogroups J and T), and IW (haplogroups I, W, X, and N1). The distribution of these four mtDNA haplogroup clusters was consistent between the patient and control cohorts (*R* × *C* test of independence; *G* = 3.933, DF = 3, *P* = 0.269; table 1). This finding is consistent with the previously observed lack of association of mtDNA haplogroups as a sole genetic component with T2DM in Caucasians (Saxena et al. 2006).

To detect candidate mtDNA–nDNA genotype combinations differing in their representation between the patients and controls, we divided the tested samples into 12 different mtDNA–nDNA genotypes (4 mtDNA lineages × 3 nDNA *NDUFC2* genotypes = 12 mtDNA–nDNA genotypes; table 2). Using a permutation test, we found that the HV–CC genotype (i.e., mtDNA haplogroup HV and the *NDUFC2* SNP genotype CC) was underrepresented in the patient group (*P* = 0.006 and *q* = 0.016; table 2). Therefore, this genotype combination (HV–CC) was used as the reference group in the logistic regression analysis designed to test whether susceptibility to developing T2DM differed among the mtDNA–nDNA genotypes. By converting the categorical variable mtDNA–nDNA genotype into a set of binary predictor variables (i.e., dummy variable), we could compare the reference mtDNA–nDNA genotype, detected using the permutation test (i.e., HV–CC) with each of the other 11 mtDNA–nDNA genotypes using a single test, while controlling for possible effects of other individual characteristics (e.g., sex and age) (table 3). Our analysis revealed that the HV–CC genotype was significantly underrepresented in T2DM patients, particularly in comparison with five mtDNA–nDNA genotypes, HV–CG, UK–CG, UK–GG, IW–CC, and JT–CC (*P* = 0.029, OR = 1.58; *P* = 0.002, OR = 2.01; *P* = 0.018, OR = 2.44; *P* = 0.033, OR = 1.98; *P* = 0.018, OR = 1.90, respectively, table 3). These results also remained significant after correction for multiple testing (*q* = 0.020, *q* = 0.006, *q* = 0.018, *q* = 0.020, *q* = 0.018, respectively). This implies that the HV–CC mitonuclear genotype likely confers a protective effect against T2DM, especially relative to certain mitonuclear genotype combinations.

**Table 3:** Logistic Regression Comparing the Propensity to Develop T2DM between Individuals Pertaining to the HV–CC genotype and Those in Each of the Other 11 mtDNA–nDNA genotypes

Variable	Coefficient (SE)	Odds Ratio (95% CI)	*P* Value	*q* Value (π_0_ = 0.275) Smoother	*q* Value (π_0_ = 0.260) Bootstrap
Constant	3.63 (0.449)		0.000		
HV_CG	0.46 (0.21)	1.58 (2.38–1.05)	**0.029**	**0.020**	**0.019**
HV_GG	0.14 (0.389)	1.15 (2.47–0.54)	0.718	0.197	0.186
UK_CC	0.23 (0.192)	1.25 (1.83–0.86)	0.243	0.088	0.083
UK_CG	0.70 (0.224)	2.01 (3.12–1.3)	**0.002**	**0.006**	**0.006**
UK_GG	0.89 (0.377)	2.44 (5.11–1.17)	**0.018**	**0.018**	**0.017**
IW_CC	0.68 (0.32)	1.98 (3.7–1.06)	**0.033**	**0.020**	**0.019**
IW_CG	−0.15 (0.277)	0.86 (1.48–0.5)	0.585	0.177	0.167
IW_GG	1.54 (1.07)	4.65 (37.86–0.57)	0.151	0.065	0.062
JT_CC	0.64 (0.27)	1.90 (3.22–1.12)	**0.018**	**0.018**	**0.017**
JT_CG	0.36 (0.321)	1.44 (2.69–0.77)	0.261	0.088	0.083
JT_GG	1.10 (0.651)	3.01 (10.77–0.84)	0.091	0.046	0.043

Note.—Variable: Model constant and the compared mtDNA–nDNA genotypes. The test compares each of the genotypes with the reference genotype (HV–CC). Coefficient (standard error, 1 SE) and odds ratio (95% confidence intervals, CI) are listed. *P* values (measuring the minimum false-positive rate, which is incurred when calling a test significant) and *q* values (measuring the minimum false discovery rate, which is incurred when calling a test significant) were estimated using the smoother method proposed in Storey and Tibshirani (2003) and the bootstrap method proposed in Storey (2002), both implanted in the QVALUE software package (Storey et al. 2004). π_0_ measures the overall proportion of true null hypotheses. Bold and underlined—significant.

## Discussion

In this work, we provided support for our hypothesis that mitonuclear interactions and coevolution are important for cell life. We focused our analyses on *NDUFC2*, an nDNA-encoded complex I subunit that directly interacts and coevolves with mtDNA-encoded subunits. We found that downregulation of *NDUFC2* in human cells decreased mitochondrial membrane potential, especially in growth conditions that prefer use of OXPHOS, and affected the integrity of complex I. Moreover, site-directed mutagenesis in the positively selected amino acid 46 of NDUFC2 significantly affected the direct interaction of NDUFC2 with the mtDNA-encoded ND4 subunit. Finally, we found that certain genotype combinations of human variants within amino acid 46 in NDUFC2 and the mitochondrial genome affected the susceptibility to develop T2DM. Overall, these combined pieces of evidence offer further support for the role of NDUFC2 in complex I protein–protein interactions, in general, and to mitonuclear interactions, in particular, and suggest that NDUFC2 is likely involved in the structural stability of complex I. These findings also lend insight into the importance of mitonuclear interactions for the stability and function of complex I.

Being one of the accessory subunits of complex I, that is, a component that was added to complex I during the course of evolution yet was not present in the bacterial ancestor, the functionality of NDUFC2 has not been well studied to date. The overall functional analysis of NDUFC2 revealed a notable decrease in the total amount of assembled complex I, which was consistent with the decreased NADH oxidation and with the significant reduction in the mitochondrial membrane potential in cells. Thus our approach demonstrates the importance of this subunit for the structural integrity of mammalian complex I and for mitonuclear interactions within the complex. Hence, our study paves the path toward investigating the functional importance of other accessory subunits of the complex, as well as their importance for the network of subunit interactions in complex I. Because complex I is the protein complex most involved in mitochondrial disorders, being vital in complex disorders, such as Parkinson’s disease (Winklhofer and Haass 2010) and oncocytomas (Zimmermann et al. 2011), deciphering the functionality of the 44 subunits of the complex and their network of interaction is of fundamental importance for understanding the molecular basis of complex I involvement in these disorders.

Our site-directed mutagenesis experiments indicate that the positively selected amino acid 46 in NDUFC2 is essential for its interaction with the mtDNA-encoded ND4 subunit. A phylogenetic analysis of NDUFC2 revealed that amino acid position 46 was mainly occupied by the following three hydrophobic amino acid residues (fig. 1): 1) alanine, which is present in the majority of the nonprimate mammals and prosimians; 2) phenylalanine, which became fixed in old world monkeys; and 3) leucine, which became fixed during the radiation of apes. Notably, the less prevalent human genetic variant of amino acid 46, replacing a leucine for a valine, apparently occurred only once during mammalian evolution. Thus, all the amino acid replacements that we performed at NDUFC2 position 46 were previously chosen during evolution and were successfully retained in living organisms. Because “swapping” of this amino acid alone led to an altered ability of human NDUFC2 to interact with its mtDNA-encoded binding partner (ND4), it is likely that in order to maintain complex I integrity during evolution, additional compensatory changes must have occurred and were selected for. This possibility is currently under investigation.

How could one explain the effect of amino acid replacement at NDUFC2 position 46 on the binding capacity of the protein? Because the amino acids that we encoded into the *NDUFC2* constructs (i.e., A, F, V, and L) are similar in their hydrophobic properties, the simplest explanation for the affinity differences would be the effect of amino acid size. Indeed, this interpretation is supported by our experiments. While introducing the largest amino acid (phenylalanine) led to decreased protein–protein interactions, the smallest amino acid tested (alanine) led to slightly increased, yet significant, levels of protein–protein interactions.

Although we showed the importance of mitonuclear coevolution and physical interactions involving NDUFC2 for the structural integrity of complex I, it was unclear how such interaction affects the phenotype of the organism. In order to evaluate this point, we took a step toward assessing the importance of mitonuclear interactions for human health. Here, we showed that a common genetic variant within *NDUFC2* altered the susceptibility of developing T2DM only in combination with a certain mtDNA genetic background (haplogroup cluster HV). This implies that both the nDNA and the mtDNA-encoded variants in this genotype combination should have functional attributes. The first support for this hypothesis came when we assessed the functionality of one participant of the T2DM-associated mitonuclear combination, the common *NDUFC2* L46V variant. Sequence analysis using SELECTON revealed that amino acid position 46 in NDUFC2 underwent positive selection during mammalian evolution, thus reflecting functional potential. Second, we performed a domain analysis of NDUFC2 and showed that this amino acid position is predicted to be involved in protein–protein interactions. Third, we showed that siRNA-mediated knock-down of *NDUFC2* affected complex I integrity. Fourth, using a modified yeast two-hybrid assay to reflect quantitative differences in protein–protein interactions while mutating amino acid 46, we determined the contribution of NDUFC2 position 46 to mitonuclear protein–protein interactions. Finally, the mtDNA haplogroup cluster HV was previously associated with altered risk to develop neurological disorders, including schizophrenia (Amar et al. 2007) and Parkinson’s disease (Hudson et al. 2013), and had a modest protective effect against AIDS progression (Guzmán-Fulgencio et al. 2013). Thus, both NDUFC2 amino acid 46 and mtDNA haplogroup cluster HV indeed have functional implications. Nevertheless, in our hands, no clear functional difference was observed between the valine and leucine nonsynonymous variants occupying NDUFC2 amino acid position 46 in humans. Because more radical amino acid substitutions that we performed revealed that NDUFC2 amino acid position 46 is functionally important, it is plausible that the leucine to valine nonsynonymous change has a more subtle effect. To assess such subtle impact, we performed a disease association study, a well-known approach designed to identify marginal phenotypic impacts of genetic variants. This study focused on T2DM, because mounting evidence supports the genetic involvement of OXPHOS in the etiology of T2DM.

Several studies have pointed out T2DM association of genetic variants both in nDNA-encoded mitochondrial genes (Krempler et al. 2002; Muller et al. 2003; Lindholm et al. 2004; Wang et al. 2004) and in mtDNA (Poulton et al. 2002; Guo et al. 2005; Mohlke et al. 2005; Fuku et al. 2007; Nishigaki et al. 2007; Feder et al. 2009; Achilli et al. 2011). However, many of these studies assessed the disease association of variants either within the nDNA or in the mtDNA, thus overlooking the impact of mitonuclear interactions. We previously showed that T2DM association with mtDNA genetic backgrounds varied among populations and depended on the health status of the parents (Feder et al. 2009), suggesting the involvement of nDNA-encoded modifying factors in the phenotypic effect of mtDNA genetic variants. Other studies indicated that the penetrance of mtDNA pathologic mutations is modulated by the nDNA genetic background (Hudson et al. 2005; Potluri et al. 2009) and vice versa (Gaweda-Walerych et al. 2010). Hence, the very basic and unique characteristics of the mitochondrial genetic system, namely, its interplay with mtDNA-encoded, nDNA-encoded, and environmental factors should be considered while investigating mitochondrial genetic involvement in T2DM, and probably in other complex phenotypes (Zhu et al. 2014). In this study, we demonstrated one aspect of this interplay, namely that association of mitochondrial genetic factors with T2DM in Caucasians occurred only when a specific mtDNA genetic background (HV) was combined with a specific *NDUFC2* nonsynonymous variant. We interpreted these results to mean that the mitonuclear genotype combination HV–CC is likely involved in the etiology of T2DM, at least in Ashkenazi Jews. This finding has a broader implication: Most successful genome-wide association studies reveal only the marginal phenotypic effect of certain genetic factors. These studies have limited statistical capacity and frequently encounter type 1 errors for the detection of contributing epistatic interactions to complex disorders (Ueki and Cordell 2012). Our study demonstrated the advantage of the candidate gene approach while searching for T2DM-associated epistatic interactions, especially because of the focus on a specific biochemical pathway with known function and strong connection to the etiology of the disease. Although encouraging, these results should be replicated in independent Ashkenazi Jewish patient and control cohorts once larger sample sizes become available.

Notably, T2DM association was observed for a single mitonuclear genotype combination involving *NDUFC2* and haplogroup HV (HV–CC). Because haplogroup HV is not defined by mutations in the mtDNA-encoded interacting partner of *NDUFC2*, *ND4*, the interference with disease association likely occurred only through the other *NDUFC2* variant, valine 46. Previously, we observed coevolution and physical interaction between NDUFC2 and ND4 proteins, involving several amino acid positions (Gershoni et al. 2010). Currently, we are investigating the importance of these coevolving amino acids for the physical interactions of NDUFC2 and ND4. Nevertheless, it is worth noting that we currently cannot rule out the attractive possibility that the observed association of mitonuclear genotype with T2DM is due to inter-OXPHOS complex interactions in the frame of OXPHOS super complexes (Lapuente-Brun et al. 2013).

In conclusion, our results underline the importance of mitonuclear interactions and coevolution in maintaining OXPHOS function and the integrity of OXPHOS complex I. We further showed that mutations in the code for a positively selected amino acid in *NDUFC2*, a nuclear DNA-encoded subunit of complex I, affected its interaction with an mtDNA-encoded subunit of the complex. This finding stresses the implication of the signature of natural selection on the function of the protein. Because *NDUFC2* was not present in the bacterial complex I and similar to other “supernumerary subunits” was added after the radiation of eukaryotes (Gabaldon et al. 2005), our results lend further support to the functional importance of the supernumerary subunits in disease (Haack et al. 2012) and in complex I integrity (Mimaki et al. 2011). Finally, we found that interfering with mitonuclear interactions, rather than with each genome alone, altered the susceptibility to develop T2DM in Ashkenazi Jews. In the future, larger samples sizes will allow increasing the resolution of such disease associations from mtDNA haplogroup clusters to haplogroups and possibly even to haplotypes. It is worth noting that the results presented here currently apply only to Ashkenazi Jews and should be confirmed in other independent populations. Better understanding of the network of subunit interactions of mammalian complex I will allow testing for the functional importance of more candidate mitonuclear interactions in this complex not only during evolution but also in health and in disease.

## Supplementary Material

Supplementary figures S1–S3 and tables S1–S4 are available at *Genome Biology and Evolution* online (http://www.gbe.oxfordjournals.org/).

Supplementary Data
