# Supplementary material for: Disrupting Mitochondrial–Nuclear Coevolution Affects OXPHOS Complex I Integrity and Impacts Human Health
Source: Genome Biol Evol. 2014 Sep 22;6(10):2665–80. doi: 10.1093/gbe/evu208 (PMC4224335; doi:10.1093/gbe/evu208)
Supplement: Supplementary Data [file supp_6_10_2665__index.html]

Disrupting mitochondrial-nuclear co-evolution affects OXPHOS complex I integrity and impacts human health — Disrupting Mitochondrial–Nuclear Coevolution Affects OXPHOS Complex I Integrity and Impacts Human Health — Supplementary Data 

# Disrupting Mitochondrial–Nuclear Coevolution Affects OXPHOS Complex I Integrity and Impacts Human Health

## Supplementary Data

files

**Files in this Data Supplement:**

- Supplementary Data - pdf file
- Supplementary Data - pdf file
- Supplementary Data - pdf file
- Supplementary Data - xlsx file
- Supplementary Data - docx file
