# Supplementary figures and images for: Disrupting Mitochondrial–Nuclear Coevolution Affects OXPHOS Complex I Integrity and Impacts Human Health
Source: Genome Biol Evol. 2014 Sep 22;6(10):2665–80. doi: 10.1093/gbe/evu208 (PMC4224335; doi:10.1093/gbe/evu208)

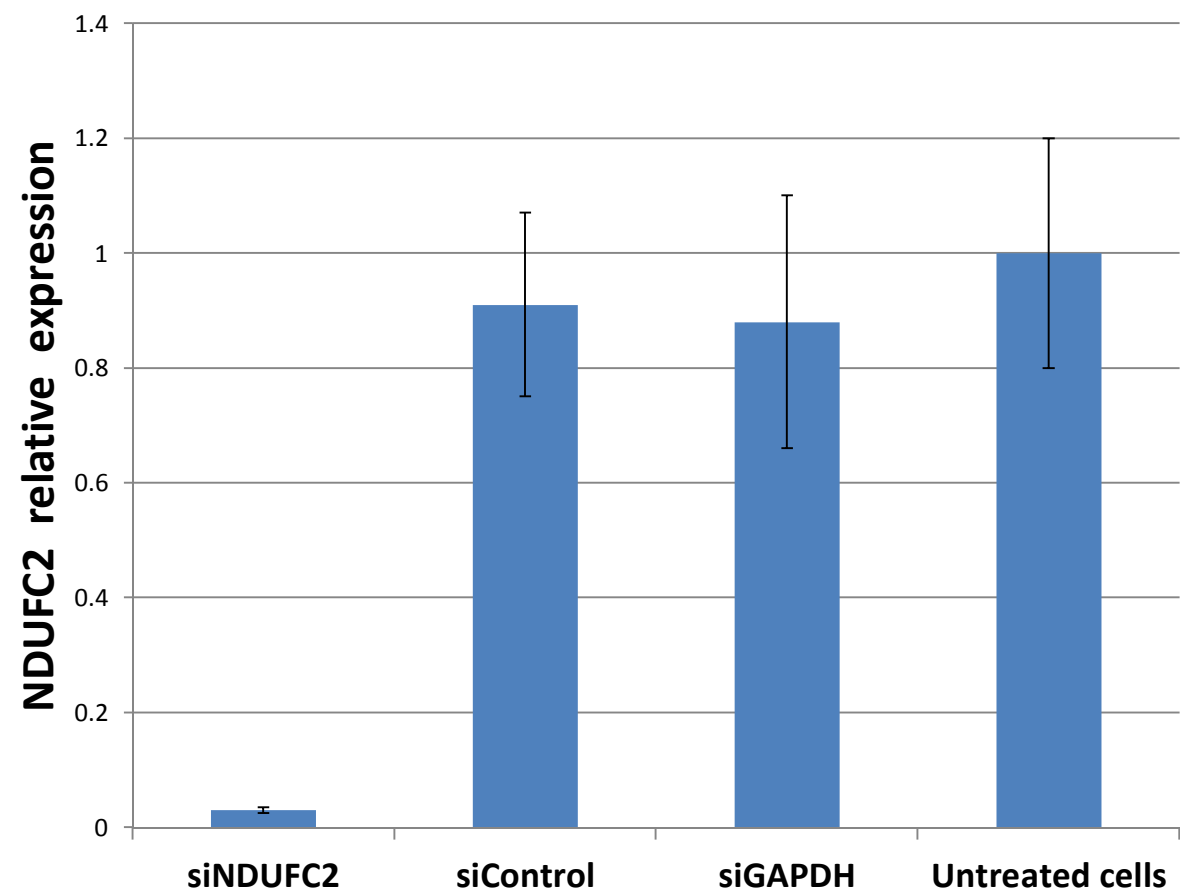

Supplement: Supplementary Data [file supp_evu208_Gershoni_2014_FigureS1_revised-new_High_resolution.pdf]

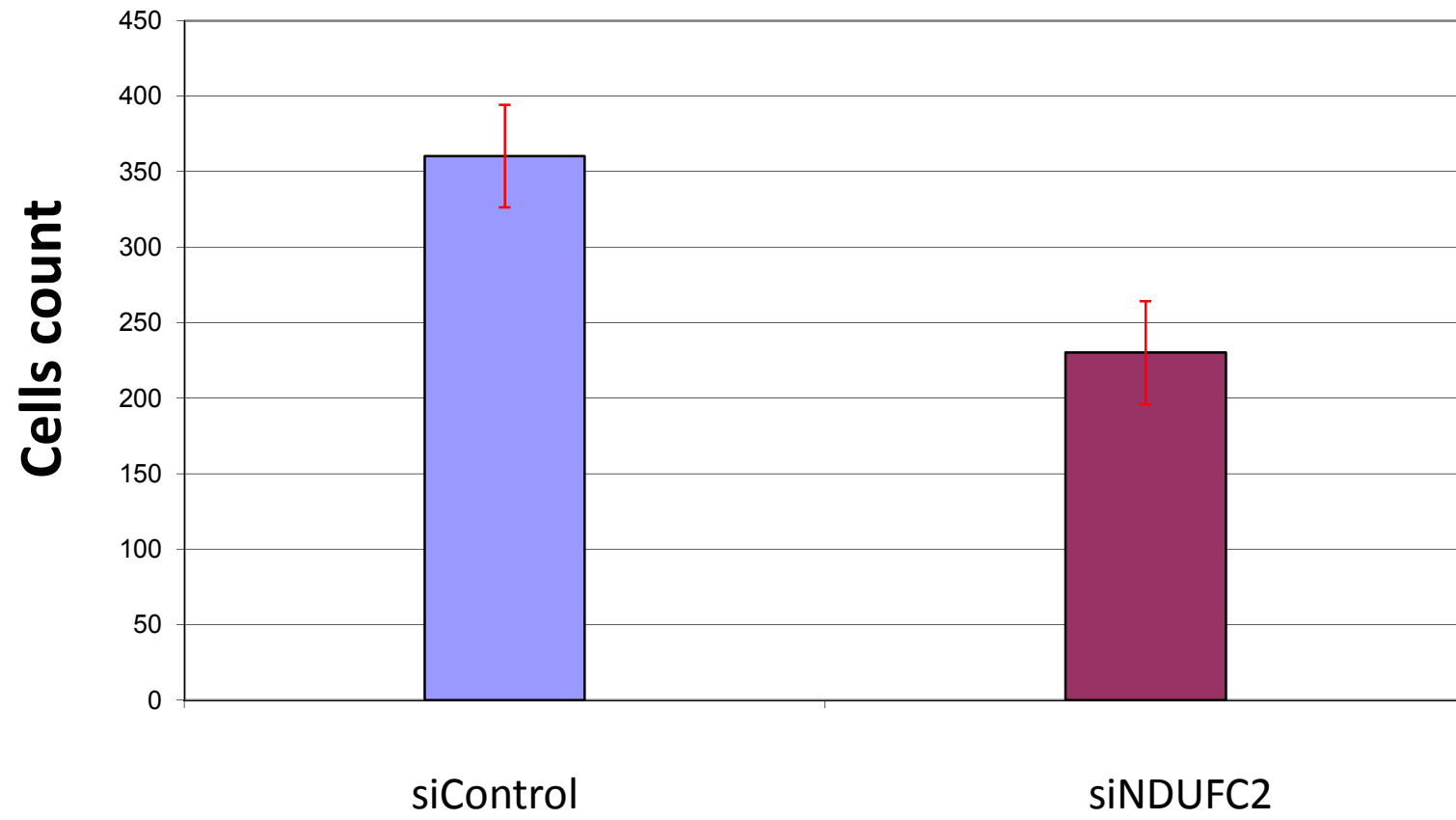

Supplement: Supplementary Data [file supp_evu208_Gershoni_2014_FigureS2_revised.pdf]

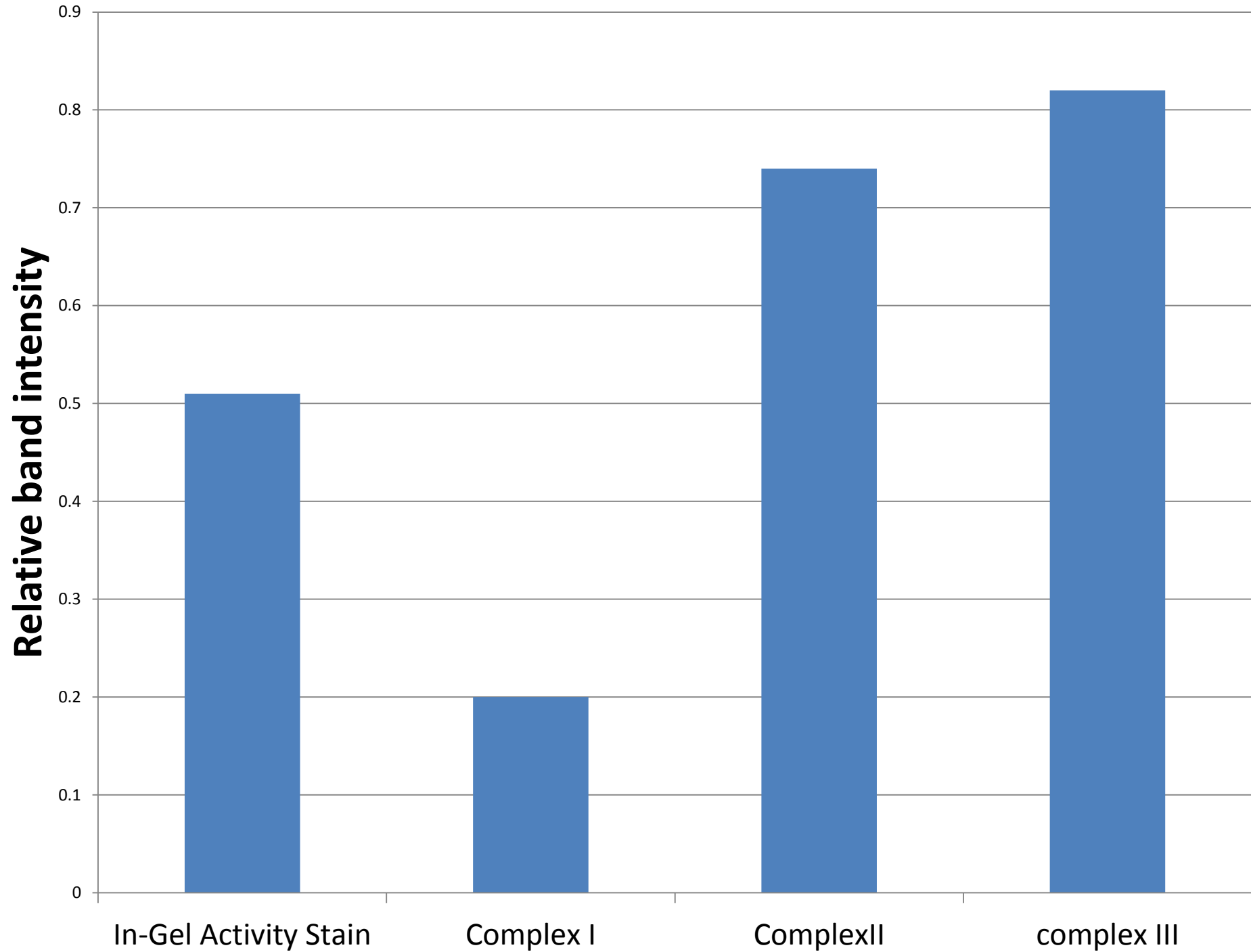

Supplement: Supplementary Data [file supp_evu208_Gershoni_2014_GBE_FigS3_densitometry_revised.pdf]
