# Supplementary material for: Disrupting Mitochondrial–Nuclear Coevolution Affects OXPHOS Complex I Integrity and Impacts Human Health
Source: Genome Biol Evol. 2014 Sep 22;6(10):2665–80. doi: 10.1093/gbe/evu208 (PMC4224335; doi:10.1093/gbe/evu208)
Supplement: Supplementary Data [file supp_evu208_Gershoni_et_al_Supplementary_material_September2014.docx]

**Supplementary information**

**Table S1 (Excel file): Summary of genotype and general phenotype information for patients and controls.** A) Patients (1118) and healthy controls (359) related information, including mitochondrial haplogroups, nuclear genotype of the SNP rs8875, gender and age. B) Medical information of 399 patients that were first used in this study. C) Summary statistics of the 399 patient's medical information including age, BMI and Waist–hip ratio divided according to gender.

**Table S2: List of 28 mtDNA SNPs in Affymetrix 6.0 chip that were informative for haplogroup assignment.**

| Probe Set ID | dbSNP | Allele A | Allele B | rCRS Nucleotide Position |
| --- | --- | --- | --- | --- |
| SNP_A-8574928 | rs28357975 | C | T | 4580 |
| SNP_A-8574539 | rs2015062 | C | T | 7028 |
| SNP_A-8574963 | rs28358887 | C | T | 8994 |
| SNP_A-8574933 | rs28358268 | A | G | 9055 |
| SNP_A-8574516 | rs9743 | A | G | 9698 |
| SNP_A-8574937 | rs28358274 | A | G | 10086 |
| SNP_A-8574938 | rs28358275 | C | T | 10238 |
| SNP_A-8574941 | rs28358279 | A | G | 10463 |
| SNP_A-8574942 | rs28358280 | C | T | 10550 |
| SNP_A-8574771 | rs3915952 | C | T | 11251 |
| SNP_A-8574943 | rs28358285 | C | T | 11299 |
| SNP_A-8574549 | rs2853493 | A | G | 11467 |
| SNP_A-8574944 | rs28358286 | C | T | 11674 |
| SNP_A-8574551 | rs2853495 | C | T | 11719 |
| SNP_A-8574741 | rs3088053 | C | T | 11812 |
| SNP_A-8574554 | rs2853498 | A | G | 12308 |
| SNP_A-8574968 | rs28359172 | C | T | 12612 |
| SNP_A-8574706 | rs2854122 | A | G | 12705 |
| SNP_A-8574764 | rs3899498 | C | T | 13368 |
| SNP_A-8574729 | rs2857287 | A | G | 13506 |
| SNP_A-8574917 | rs28357671 | C | T | 14178 |
| SNP_A-8574756 | rs3135030 | C | T | 14470 |
| SNP_A-8574920 | rs28357682 | C | T | 14905 |
| SNP_A-8574921 | rs28357684 | C | T | 15043 |
| SNP_A-8575338 | rs28573847 | C | T | 15301 |
| SNP_A-8574742 | rs3088309 | G | T | 15452 |
| SNP_A-8574911 | rs28357372 | A | G | 15607 |
| SNP_A-8574687 | rs2853510 | A | G | 15924 |

**Table S3. List of primers for the amplification of all *NDUFC2* exons.**

| **Gene name** | **Amplified Fragment** | **primer name** | **sequence** |
| --- | --- | --- | --- |
| NDUFC2 | exon 1 | NDUFC2ex1F | 5' GCA AAG TGA AAC TTA GTA GC -3' |
| NDUFC2 | exon 1 | NDUFC2ex1R | 5' TTC ACA GAC TCA GTT CCT CG -3' |
| NDUFC2 | exon 2 | NDUFC2ex2F | 5' GAT GCT GAC ATC ATC ATC TCG AAC GC -3' |
| NDUFC2 | exon 2 | NDUFC2ex2R | 5' GTG GAC AGT AGT GCC ATT CTC TGA AG -3' |
| NDUFC2 | exon 3 | NDUFC2ex3F | 5' CAT GAA CAT TCA GAC CAC AGC -3' |
| NDUFC2 | exon 3 | NDUFC2ex3R | 5' CAG AAA CAG CAG GTA TCA GTG -3' |

The primers were designed using the following NCBI reference sequences: NDUFC2: NC_000011.7.

**Table S4. PCR conditions for the exons amplification of *NDUFC2*.**

| Comments | Mg concentration | DMSO addition | Amp. (72^o^C) | Annealing | Length of fragment | Primer name | Gene name |
| --- | --- | --- | --- | --- | --- | --- | --- |
| - | 2mM | + | 1 Min | 55 ^o^C, 30 Sec | 623 bp | NDUFC2ex1 | NDUFC2 |
| - | 1.5mM | - | 1 Min | 62 ^o^C, 30 Sec | 934 bp | NDUFC2ex2 |  |
| 50 PCR cycles | 1.5mM | - | 1 Min | 57 ^o^C, 30 Sec | 413 bp | NDUFC2ex3 |  |

**Figure S1: *NDUFC2*-silencing efficiency.** D-407 cells were transformed with oligonucleotide against either *NDUFC2* or *GAPDH,* as a positive control for the silencing system. Cells transformed with a non-targeting oligonucleotide served as a negative control as did untreated cells. X axis: *NDUFC2* transcript levels are presented in columns after treatment with *NDUFC2* siRNA (siNDUFC2), treatment with non-targeting oligonucleotide (siControl), treatment with GAPDH siRNA (siGAPDH) and in untreated cells. Error bars are the result of three independent experiments. Y-axis: percentage of *NDUFC2* transcript levels in each experiment as compared to untreated cells. Transcript levels were measured by real time PCR in each experiment as compared to untreated cells (right column) representing 100% expression (1.0). The results show 90% knock-down in *NDUFC2* expression only upon transformation with oligonucleotides against *NDUFC2* (siNDUFC2).

**Figure S2: *NDUFC2*-silencing affects cell growth.** Cell counts (standard counting chamber) were obtained from transient *NDUFC2* knock-down experiments in the human D-407 retinal pigment epithelium cell line (see Materials and methods section). X-axis: cell counts in (siNDUFC2) *NDUFC2*-silenced cells and in (siControl) cells treated with non-targeting siRNA agent (negative control). Error bars are the result of three independent experiments. Y axis: mean cell counts in a standard counting chamber. A significance decrease in the growth capability of knocked-down cells was observed (t-test, p= 0.003).

**Figure S3: Band intensity measurements of OXPHOS complexes and complex I in-gel activity stain in *NDUFC2* knockout and untreated cells.** The optical density of each lane was measured using the gel analysis option of the ImageJ software (imagej.nih.gov/ij/). X axis: the tested OXPHOS complex or in-gel complex I activity stain. Y-axis: ratios between the optical density of the tested OXPHOS complexes observed in *NDUFC2* knock down cells (siNDUFC2) divided by that observed in the non-targeting knock down cells (control). The results reveal > 80% decrease in complex I (CO I) staining and 50% decrease of the in-gel activity staining suggesting significant decrease in the amount of assembled complex I and its activity.
